# Anesthesia for Cesarean Section in Parturients with Abnormal Placentation: A Retrospective Study

**DOI:** 10.7759/cureus.5033

**Published:** 2019-06-29

**Authors:** Orhan Binici, Evren Büyükfırat

**Affiliations:** 1 Anesthesiology and Critical Care, University of Harran, Sanliurfa, TUR

**Keywords:** cesarean section, placenta disorders, general anesthesia, spinal anesthesia

## Abstract

Introduction

Placental invasion anomalies are associated with high mortality and may require hysterectomy due to the high risk of massive hemorrhage. The aim of this retrospective study was to evaluate intraoperative anesthetic management, postoperative follow-up, clinical features, and fetal wellbeing in patients undergoing cesarean section due to placental invasion anomalies in a tertiary health center.

Methods

The retrospective study included patients that underwent cesarean section due to placental invasion anomalies at a tertiary health center over the period between 2013 and 2018. Intraoperative anesthetic management, blood and blood products transfusion, and total volume of blood loss, as well as neonatal Apgar score and postoperative intensive care unit (ICU) follow-up, were reviewed for each patient.

Results

The study evaluated a total of 92 patients that underwent cesarean section due to placental invasion anomalies, including 49 patients with placenta previa, 42 patients with placenta percreta, and one patient with placenta accreta. Of the 92 patients, 59 (64.1%) patients underwent general anesthesia, 31 (33.7%) underwent spinal anesthesia, and two (2.2%) underwent spinal anesthesia followed by general anesthesia. Hysterectomy was performed in four patients, including three patients who underwent general anesthesia and one patient who started with spinal anesthesia and subsequently switched to general anesthesia prior to a hysterectomy. The Apgar scores at min 1 and min 5 after the induction of anesthesia were significantly lower in patients who underwent general anesthesia as compared to those who underwent spinal anesthesia (p=0.002 and p=0.007, respectively). The duration of surgery and intraoperative blood loss were significantly higher in patients with placenta percreta as compared to other patients (p<0.001 for both).

Conclusion

In surgical planning for the patients with placental invasion anomalies, care should be taken by anesthesiologists to select the most ideal anesthetic technique, by taking into account the type of anomaly, probable volume of blood loss, and surgical complications, to ensure both maternal and fetal wellbeing. Moreover, the coordination of a team of well-educated and experienced staff is essential.

## Introduction

The anesthetic management of patients with placental invasion anomalies is highly important due to the prolonged duration of surgery, requirement for additional interventions, and disruption of hemodynamics as a result of massive hemorrhage. Placenta previa is defined as placenta implanted in the lower segment of the uterus. Prevalence of placenta previa has recently increased and accounts for approximately 0.5% of all pregnancies. This relative increase correlates with the increasing rates of cesarean section. Increasing placenta previa and cesarean section may cause placental invasion anomalies. Placental invasion anomalies can be in three forms. Placenta accreta, in which the villi adhere to the surface of the myometrium, placenta increta, in which the villi penetrate the myometrium, and placenta percreta, in which the villi penetrate through the uterine serosa and sometimes into neighboring organs such as cervix, bladder, or bowel [1,2-3].

In surgical planning for patients with placental invasion anomalies, care should be taken to select the most ideal anesthetic technique due to the prolonged duration of surgery, massive hemorrhage, requirement of hysterectomy, and an increased risk of postoperative intensive care unit (ICU) admission. Moreover, anesthesiologists should consider not only maternal but also fetal wellbeing when determining the anesthetic technique. In the present study, we aimed to retrospectively evaluate the demographic characteristics, intraoperative anesthetic management, postoperative follow-up, clinical features, intraoperative fluid management, and fetal wellbeing in patients who underwent cesarean section in our clinic due to placental invasion anomalies.

## Materials and methods

This retrospective study included 92 patients that underwent cesarean section due to placental invasion anomalies at Harran University Medical School Hospital over the period between 2013 and 2018. Patient records were retrieved from the hospital database and were reviewed for each patient.

Age, number of pregnancies, gestational age, number of previous cesarean sections, intraoperative anesthetic management, American Society of Anesthesiologists (ASA) scores, preoperative hemoglobin (Hgb) and hematocrit (Hct) values, duration of surgery (min), perioperative blood loss (mL), and neonatal Apgar scores were recorded for each patient. Moreover, intraoperative red blood cells (RBC) (unit), fresh-frozen plasma (unit), tranexamic acid (transamin 10%) human fibrinogen concentrates (Haemocomplettan P), crystalloid (mL), and colloid (mL) transfusions were also reviewed. Additionally, postoperative follow-up and length of ICU stay were also noted for each patient. The requirement of cesarean hysterectomy and the type of placental invasion anomaly were recorded based on the surgical notes.

Statistical analysis

Data were analyzed using SPSS for Windows 23.0 (IBM Corp., Armonk, NY, US). As the number of cases in the study and the number of cases in each group were remarkably low, continuous variables were expressed as median (minimum-maximum) and categorical variables were expressed as frequencies and percentages. Moreover, due to the low number of cases, pairwise comparisons were performed using the Mann-Whitney U test. Categorical variables were compared using the chi-square test. A p-value of <0.05 was considered significant.

## Results

Over the period between 2013 and 2018, cesarean section was performed in a total of 5,788 patients in our hospital. Of these, 92 patients were included in the study since they had complete medical records.

The ages of the patients ranged between 20 and 44 (mean 33) years. Of the 92 patients, 49 were detected with placenta previa, 42 with placenta percreta, and one with placenta accreta. Of the 92 patients, 59 (64.1%) underwent general anesthesia, 31 (33.7%) underwent spinal anesthesia, and two (2.2%) underwent spinal anesthesia followed by general anesthesia. General anesthesia was performed in 26 patients with placenta previa and in 32 patients with placenta percreta. Spinal anesthesia was performed in 23 patients with placenta previa and in eight patients with placenta percreta (Tables 1-2).

**Table 1:** Comparison of patients with placental invasion anomalies ES: Erythrocyte suspension, ICU: Intensive care unit

				Diagnosis				p
				Total	Placenta previa	Placenta percreta	Placentaaccreta	
				(n=92)	(n=49)	(n=42)	(n=1)	
				n(%) / median (min-max)				
Age				33 (20-44)	33 (20-44)	33 (23-42)	32	0.505*
Pregnancy count				5 (1-11)	5 (1-11)	5 (2-10)	4	0.596*
Number of previous cesarean sections				2 (0-6)	1 (0-5)	3 (0-6)	3	<0.001*
Operation time (min)				100 (45-250)	80 (45-150)	125 (65-250)	85	<0.001*
Bleeding amount (mL)				550 (250-3000)	400 (250-850)	875 (350-3000)	500	<0.001*
Crystalloid								
	1500 cc below			18 (19.6%)	18 (36.7%)	-	-	
	1500 cc -3000 cc			42 (45.7%)	19 (38.8%)	22 (52.4% )	1	
	3000 cc -5000 cc			24 (26.1%)	8 (16.3%)	16 (38.1%)	-	0.001**
	5000 cc over			8 (8.7%)	4 (8.2%)	4 (9.5%)	-	
Colloid								
	Not give			60 (65.2%)	39 (79.6%)	20 (47.6%)	1	
	500 cc			25 (27.2%)	8 (16.3%)	17 (40.5%)	-	0.003**
	1000 cc			7 (7.6%)	2 (4.1%)	5 (11.9%)	-	
intraoperative ES (unit)								
Not give				47 (51.1%)	31 (63.3%)	15 (35.7%)	1	
≤3 unit				39 (42.4%)	15 (30.6)	24 (57.1%)	-	0.028**
>3 unit				6 (6.5%)	3 (6.1%)	3 (7.1%)	-	
Fresh-frozen plasma (unit)								
Not give				69 (75%)	43 (87.8%)	25 (59.5%)	1	
≤3 unit				21 (22.8%)	5 (10.2%)	16 (38.1%)	-	0.006**
>3 unit				2 (2.2%)	1 (2%)	1 (2.4%)	-	
Need for postoperative ICU								
Yes				18 (19.6%)	3 (6.1%)	15 (35.7%)	1	<0.001**
No				74 (80.4%)	46 (93.9%)	27 (64.3%)	-	
Length of ICU stay (day)				0 (0-5)	0 (0-5)	0 (0-5)	-	0.003*
Type of anesthesia								
		General anesthesia		59 (64.1%)	26 (53.1%)	32 (76.2%)	1	0.101**
		Spinal anesthesia		31 (33.7%)	23 (46.9%)	8 (19%)	-	
		General+spinal anesthesia		2 (2.2%)	-	2 (4.8%)	-	
			*Mann-Whitney U test, **Chi-square test					

**Table 2:** Comparison of patients according to type of anesthesia ICU: Intensive care unit; ASA: American Society of Anesthesiologists

				TypeAnesthesia					P
				General Anesthesia		Spinal Anesthesia		General & Spinal Anesthesia	
				(n=58)		(n=31)		(n=2)	
				n(%) / median (min-max)					
Age				34 (23-44)		33 (20-44)		29,5 (26-33)	0.750*
Pregnancy count				5 (1-11)		5 (1-11)		5.5 (4-7)	0.475*
Number of previous cesarean sections				2 (0-6)		1 (0-5)		3,5 (3-4)	0,009*
ASA score									
	1			5 (8.6%)		5 (16.1%)		-	
	2			41 (70.6%)		25 (80.6%)		1	
	3			12 (20.6%)		1 (32%)		1	0,028**
Operation time (min)				109 (50-250)		75 (45-240)		117.5 (105-130)	<0,001*
Bleeding amount (mL)				600 (300-3000)		350 (250-1800)		1775 (1250-2300)	<0,001*
APGAR 1 minute				6 (0-8)		7 (0-8)		8 (8-8)	0,002*
APGAR 5 minute				8 (0-10)		9 (0-10)		10 (10-10)	0,007*
intraoperative ES (unit)									
		Not give			22 (37.9)	24 (77.4%)	-		
		≤3 Unit			30 (51.7%)	7 (22.6%)	2 (100%)		<0,001**
		>3 Unit			6 (10.3%)	-	-		
Tranexamic acid and human fibrinogen concentrates									
			Yes		26 (44.8%)	7 (22.6%)	1 (50%)		0,031**
			No		32 (55.2%)	24 (77.4%)	1 (50%)		
Need for postoperative ICU									
		Yes			15 (25.8%)	3 (9.6%)	1 (50%)		0,029**
		No			43 (74.1%)	29 (93.5%)	1 (50%)		
Length of ICU stay (day)					0 (0-5)	0 (0-4)	1 (0-2)		0.029*
Hysterectomy									
			Performed		3 (5.2%)	-	1 (50%)		0.549**
			Notperformed		55 (94.8%)	31 (100%)	1 (50%)		
*Mann-Whitney U test, **Chi-square test									

The number of previous cesarean sections was significantly higher in patients with placenta percreta as compared to other patients (p<0.001). Similarly, the duration of surgery and intraoperative blood loss were also significantly higher in patients with placenta percreta as compared to other patients (p<0.001 for both) (Table 1).

In patients with placenta percreta, the incidence of intraoperative red blood cell (RBC), fresh-frozen plasma, and crystalloid and colloid transfusions, the need for postoperative ICU admission and the length of ICU stay were significantly higher as compared to those of other patients (p=0.028, p=0.006, p=0.001, p=0.003, p=0.001, and p=0.003, respectively). In particular, the incidence of crystalloid and colloid transfusions was higher in patients with placenta percreta as compared to those of other patients (p=0.001 and p=0.003, respectively) (Table 1).

In patients who underwent general anesthesia, the duration of surgery was significantly longer and intraoperative blood loss was significantly higher as compared to those of other patients (p<0.001); the Apgar scores at min 1 and min 5 after the induction of anesthesia were significantly lower as compared to patients who underwent spinal anesthesia (p=0.002 and p=0.007, respectively) (Table 2); and the incidence of RBC transfusion, the need for postoperative ICU admission, and the length of postoperative ICU stay were significantly higher as compared to those of other patients (p<0.001, p=0.029, and p=0.029, respectively) (Table 2).

## Discussion

Placental invasion anomalies may lead to high morbidity and mortality, depending on the surgical procedure applied. Moreover, morbidity and mortality may be even higher when hysterectomy becomes mandatory in patients operated on under emergency conditions due to placental invasion anomalies [4]. During the surgical management of these anomalies, intraoperative blood loss may entail significant blood transfusion. Moreover, intraoperative complications, such as disseminated intravascular coagulation, fluid overload, acute respiratory distress syndrome, and infection, as well as hemodynamic disturbances that may necessitate postoperative ICU admission may threaten the health of both the mother and the fetus. Therefore, anesthetic management in such patients should be well-planned [5]. The risk of placental invasion anomalies is higher in the patients with a history of cesarean section, multiparity, myomectomy, endometrial curettage, and submucosal myoma and in pregnant women aged over 35 years [6]. In our study, the mean age of the patients was 33 years and 38 out of 92 patients were aged 35 years or over. Moreover, the mean number of pregnancies was five and the mean number of previous cesarean sections was two. These findings indicate that the risk factors for placental invasion anomalies detected in our patients, such as maternal age, multiparity, and the number of previous cesarean sections, were consistent with those reported in the literature.

Placenta percreta leads to a high risk of postoperative ICU admission and is associated with high morbidity due to infection, adjacent organ injury, and massive blood loss caused by the invasion of the bladder that necessitates significant blood transfusion [7-10]. In our study, the incidence of cesarean section was higher in patients with placenta percreta as compared to other patients (p<0.001). Similarly, duration of surgery, intraoperative blood loss, incidence of intraoperative RBC and fresh-frozen plasma transfusions, postoperative ICU admission, and length of ICU stay were significantly higher in patients with placenta percreta as compared to those in other patients (p<0.001, p=0.028, p=0.006, p<0.001, and p=0.003, respectively).

In addition to the higher intraoperative blood loss and longer duration of surgery in patients with placenta percreta, the incidence of crystalloid and colloid transfusions was also significantly higher in these patients as compared to other patients (p=0.001 and p=0.003, respectively). Similarly, the incidence of tranexamic acid and human fibrinogen concentrate administration in patients with placenta percreta (18 out of 42 patients) was also higher than that of other patients, though not significantly.

Literature indicates that regional anesthesia could be a better option than general anesthesia in patients undergoing surgery due to placenta previa since the inhaled anesthetics used for the induction of general anesthesia lead to a relaxation in the uterus and thereby lead to higher blood loss and a higher need for blood transfusion [11-12]. A previous study evaluated the surgical treatment of patients with placenta accreta and reported that regional anesthesia decreased intraoperative blood loss by means of a sympathetic blockage [13-14]. Another study retrospectively evaluated a total of 122 patients with placental invasion anomalies over an 18-year period and suggested that a neuroaxial block can be successfully performed in such patients. The authors also noted that patients undergoing cesarean hysterectomy can be switched to general anesthesia as needed [15]. In a similar study, the authors retrospectively evaluated the anesthetic techniques performed in the surgical treatment of 96 patients with placental invasion anomalies, suggested that neuraxial blocks can be performed in such patients, and emphasized that general anesthesia should be preferred in patients with placenta percreta [16]. A retrospective study conducted in the UK evaluated 40 patients who underwent treatment due to placental invasion anomalies and reported that 38 surgical procedures were initiated under neuraxial anesthesia and 17 of them were switched to general anesthesia [17]. Lilker et al. [18] retrospectively evaluated patients with placenta accreta and concluded that most patients can tolerate both prolonged surgery as well as significant blood loss under epidural anesthesia.

In our study, the ASA score was higher in patients who underwent spinal anesthesia as compared to that of other patients (p=0.028) and all the patients at high risk underwent neuroaxial anesthesia. Moreover, the maximum blood loss was 3,000 mL in patients who underwent general anesthesia as opposed to 1,800 mL in patients who underwent spinal anesthesia. Of the patients that underwent spinal anesthesia, however, only seven (22.6%) of them required transfusion of three or fewer units of RBC while none of them required transfusion of more than three units. In contrast, among the patients who underwent general anesthesia, 30 (51.7%) required transfusion of three or fewer units of RBC and six (10.3%) of them required transfusion of more than three units. In addition, the two patients who underwent both spinal and general anesthesia required transfusion of three or fewer units of RBC. On the other hand, the incidence of RBC transfusion and the administration of tranexamic acid and human fibrinogen concentrate were significantly higher in patients who underwent general anesthesia compared to those of other patients (p<0.001).

In our study, all patients with a high risk of hemorrhage underwent general surgery except for two patients who initially received spinal anesthesia and were subsequently switched to general anesthesia due to the disruption of hemodynamics caused by hemorrhage. Moreover, spinal anesthesia was not performed in any of the patients with a high risk of hemorrhage and in any of the patients who needed a transfusion of three or more units of RBCs.

Neonatal depression increases with the depth of general anesthesia. Moreover, the prolongation of the time from uterine incision to delivery leads to lower neonatal Apgar scores, resulting in a more acidotic neonate. In contrast, regional anesthesia has a lower impact on both fetal depression and neonatal Apgar scores as compared to general anesthesia, although it may lead to lower Apgar scores in cases with prolonged maternal hypotension [19-22]. In the present study, as consistent with the literature, the Apgar scores at min 1 and min 5 after the induction of anesthesia were significantly lower in patients who underwent general anesthesia as compared to patients who underwent spinal anesthesia (p=0.002 and p=0.007, respectively).

Regional anesthesia is known to have a lower risk of postoperative ICU admission in high-risk patients as compared to general anesthesia [23]. In our study, the need for postoperative ICU admission and the length of postoperative ICU stay were higher in patients who underwent general anesthesia as compared to other patients (p=0.029 and p=0.029, respectively). Moreover, postoperative ICU follow-up was not needed in 93.5% (n=29) of the patients who underwent spinal anesthesia and in 50% (n=1) of the patients that underwent spinal anesthesia followed by general anesthesia. These findings could be attributed to the induction of general anesthesia in patients with a high risk of hemorrhage and in patients anticipated to have a prolonged duration of surgery.

Murata et al. [24] reported a patient who was scheduled for cesarean section due to a diagnosis of placenta accreta, for whom they initially administered epidural anesthesia and had to switch to general anesthesia prior to the administration of hysterectomy due to iliac artery occlusion. Another study evaluated 65 patients who underwent surgery with a prediagnosis of placenta previa and reported that 16 out of 21 patients who were detected with placental invasion anomalies underwent general anesthesia [25]. Markley et al. [15] evaluated 129 patients who underwent cesarean section due to placenta previa and reported that 122 patients underwent neuraxial anesthesia, 15 of whom underwent a hysterectomy after switching to general anesthesia. The authors also noted that hysterectomy was the main reason for switching to general anesthesia. In our study, hysterectomy was performed in four patients, including three who underwent general anesthesia and one who started with spinal anesthesia and subsequently switched to general anesthesia prior to hysterectomy. Meaningfully, hysterectomy was not performed solely under spinal anesthesia in any patient.

## Conclusions

Due to their complications, placental invasion anomalies threaten the health of both mother and fetus. Therefore, anesthesiologists should consider not only maternal but also fetal wellbeing when determining the anesthetic technique. In the surgical treatment of patients with placental invasion anomalies, the coordination of a team of well-educated and experienced staff, as well as the preparation of blood products and essential fluids prior to surgery, are very essential. Moreover, to ensure fetal wellbeing, measures should be taken to prevent maternal hypotension such as by administering a neuraxial block in the mother, by switching to general anesthesia after the induction of neuraxial anesthesia, particularly in patients with placenta percreta or in patients scheduled for hysterectomy, or by administering general anesthesia prior to the initiation of the surgery.
